# Vaccination prevents IL-1β-mediated cognitive deficits after COVID-19

**DOI:** 10.21203/rs.3.rs-3353171/v1

**Published:** 2023-09-15

**Authors:** Abigail Vanderheiden, Jeremy Hill, Xiaoping Jiang, Ben Deppen, Gayan Bamunuarachchi, Nadia Soudani, Astha Joshi, Matthew D. Cain, Adrianus C.M. Boon, Robyn S. Klein

**Affiliations:** 1 Center for Neuroimmunology and Neuroinfectious Diseases, Washington University School of Medicine, St. Louis, MO, USA; 2 Department of Medicine, Washington University School of Medicine, St. Louis, MO, USA; 3 Department of Pathology and Immunology, Washington University School of Medicine, St. Louis, MO, USA; 4 Department of Neurosciences, Washington University School of Medicine, St. Louis, MO, USA

## Abstract

Up to 25% of SARS-CoV-2 patients exhibit post-acute cognitive sequelae. Although millions of cases of COVID-19-mediated memory dysfunction are accumulating worldwide, the underlying mechanisms and how vaccination lowers risk are unknown. Interleukin-1, a key component of innate immune defense against SARS-CoV-2 infection, is elevated in the hippocampi of COVID-19 patients. Here we show that intranasal infection of C57BL/6J mice with SARS-CoV-2 beta variant, leads to CNS infiltration of Ly6C^hi^ monocytes and microglial activation. Accordingly, SARS-CoV-2, but not H1N1 influenza virus, increases levels of brain IL-1β and induces persistent IL-1R1-mediated loss of hippocampal neurogenesis, which promotes post-acute cognitive deficits. Breakthrough infection after vaccination with a low dose of adenoviral vectored Spike protein prevents hippocampal production of IL-1β during breakthrough SARS-CoV-2 infection, loss of neurogenesis, and subsequent memory deficits. Our study identifies IL-1β as one potential mechanism driving SARS-CoV-2-induced cognitive impairment in a new murine model that is prevented by vaccination.

## Introduction

Severe acute respiratory syndrome coronavirus 2 (SARS-CoV-2) is a betacoronavirus that causes coronavirus infectious disease 2019 (COVID-19), a severe respiratory illness characterized by fever, shortness of breath, anosmia, headache, and sometimes is fatal^1,2^. Current estimates suggest 15–60% of survivors develop post-acute neurologic symptoms of COVID-19 (neuroPASC)^3–7^. Symptoms include new daily headaches, peripheral neuropathy, anosmia, anxiety, memory impairments, and lack of concentration^8–11^. Longitudinal studies have found that even mild COVID-19 is associated with decreases in total brain volume, gray matter thinning, and poor performance on cognitive tests^12–15^. Post-mortem studies indicate that productive infection of the central nervous system (CNS) does not occur in the vast majority of COVID-19 cases (Reviewed in^16,17^). Despite the absence of CNS infection, patients have evidence of microglial activation, inflammatory cytokine production (IL-6, IL-1β, TNF, Type 1 IFNs), blood-brain barrier (BBB) disruption, and T cell infiltration into the brain parenchyma^18–23^. However, the mechanisms leading to persistent neurological dysfunction are incompletely understood.

The hippocampus is essential for learning and memory and contains part of a tri-synaptic circuit that orchestrates memory formation via signals between the entorhinal cortex, the dentate gyrus (DG) region, the cornu ammonis region 3 (CA3) and the CA1^24–26^. Additionally, the sub-granular zone (SGZ) of the DG is one of two sites in the brain where adult neurogenesis occurs. Neural stem cells (NSCs) in the SGZ differentiate into Type 2 intermediate neural progenitors, then neuroblasts, which generate immature neurons, and finally become mature neurons^27^. Adult neurogenesis is critical for learning, as newly formed neurons integrate into local circuits in the DG and play an essential role in the formation of new memories^27^. Post-mortem analyses of the CNS of COVID-19 patients previously demonstrated significantly decreased adult hippocampal neurogenesis during acute infection^18^. Many pro-inflammatory cytokines, including interleukin-1 Beta (IL-1β), can inhibit neurogenesis^28,29^. In models of neuroinvasive viral infections, IL-1R1 signaling within NSCs promoted neurotoxic astrogenesis at the expense of neurogenesis, which was associated with lack of synapse recovery and deficits in spatial memory^30,31^. High levels of IL-1β in the serum are associated with increased risk of neuroPASC and immunohistochemical (IHC) analysis of post-mortem COVID-19 samples revealed elevated levels of IL-1β within hippocampal myeloid cells compared to healthy controls^18,30–32^. Whether increased IL-1β production in the hippocampus during COVID-19 inhibits adult neurogenesis and underlies memory/learning deficits is unknown.

One of the few parameters shown to reduce the risk of long-COVID/PASC is prior vaccination^33^. Breakthrough infection after vaccination is associated with decreased pro-inflammatory serum cytokines (IL-6, IFNγ, IL-1β) and limited changes in peripheral blood mononuclear cells compared to SARS-CoV-2 infected, unvaccinated individuals^34–36^. However, whether vaccination also exerts these effects in the brain after breakthrough SARS-CoV-2 infection has not been investigated. To examine mechanisms of neuroPASC and the impact of vaccination, we developed a neuroPASC model using the Beta variant (B.1.351) of SARS-CoV-2, which produces robust natural infection of the respiratory tract of C57Bl/6J mice and a lung immune response similar to that observed in humans^37–39^. Although B.1.351 infection of mice does not lead to viral neuroinvasion, it induces transient infiltration of Ly6C^high^ monocytes into the brain parenchyma and persistent microglial/macrophage activation, with elimination of hippocampal synapses. Increased IL-1β produced by monocytes and microglia inhibits adult neurogenesis, leading to memory deficits in recovered animals. Importantly, we demonstrate that even a single, intranasal, low dose of a chimpanzee-adenoviral vectored COVID-19 vaccine containing the pre-fusion stabilized SARS-CoV-2 Spike protein prevents IL-1β-mediated hippocampal dysfunction after breakthrough infection.

## Results

### Peripheral B.1.351 infection causes memory deficits in C57Bl/6J mice.

**Figure 1:**
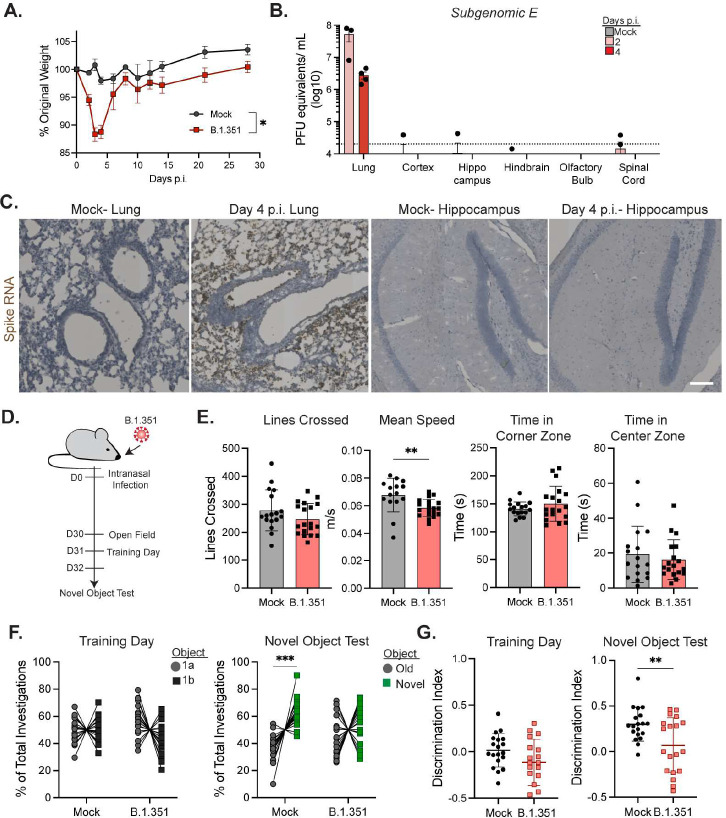
Respiratory B.1.351 infection causes memory deficits in C57Bl/6J mice. 14–16 week old C57Bl/6J mice were intranasally infected with 5 × 10^5^ pfu of B.1.351 or mock infected with PBS and weight was monitored until day 30 p.i. A) Percent of original weight for mock vs B.1.351 infected mice (n=20). B) SARS-CoV-2 Subgenomic E RNA from the indicated tissue at 2 or 4 dpi or from mock mice at 4 dpi (n=4). C) Representative images of *In situ* hybridization for SARS-CoV-2 Spike RNA (Brown) counterstained with hematoxylin (blue) from the lung and hippocampus of mice at 4 dpi. D) Experimental schematic for behavioral testing; OFT on 30 dpi, followed by a training day for the NOR test, then on 32 dpi the NOR test. E) Indicated measurements from OFT at 30 dpi (n=20 mice per group). F) Percentage of the total investigations (nose poke of the object) on NOR training day (identical Object 1a and 1b) and test day (old vs novel object). Individual mice are connected with a line (n=20). I) Discrimination indices ((# of investigations of Novel object- # investigations old object/(Total # investigations)) for the training day and test day (n=20). Data is represented as mean with standard error of mean (SEM) and was pooled from 2–3 individual experiments. Scale bar is 40 μM. Statistical significance was determined using a one-way ANOVA, two-way ANOVA, student’s t-test, or paired two-way ANOVA (for H). *=p<0.05, **=p<0.01, ***=p<0.001.

While the original Wuhan SARS-CoV-2 does not infect mice, some variants, including B.1.351 (Beta), contain sequence changes in the receptor binding domain (RBD) of the Spike protein, such as N501Y, which allow binding to mouse ACE-2^38,39^. Previous work demonstrated that B.1.351 infects the respiratory tract of C57Bl/6J mice and causes disease, although thorough characterization of viral tropism and post-acute immune responses are lacking^37–39^. After intranasal (i.n.) B.1.351 (5 × 10^5^ plaque forming units (PFU)) infection, C57Bl/6J mice (14–16 week-old) lose ~15% of their total body weight at 3–4 days post infection (dpi), followed by recovery to their original weight by 7–8 dpi (Fig. 1a). Disease severity (measured via weight loss) is age-dependent, with 8-week old B.1.351-infected mice losing <5% of their body weight and 20-week old mice losing up to 20% body weight (Suppl. Fig. 1a). Sex did not significantly impact weight loss (Suppl. Fig. 1b). Thus, we utilized 14–16 week old mice of both sexes in our study.

We investigated the kinetics and tropism of B.1.351 infection in C57Bl/6J mice via plaque assay; levels of infectious virions within lungs and nasal turbinates peak at 2 dpi, remain high until 6 dpi, and become undetectable by 12 dpi (Suppl. Fig. 1c). Plaque assay analyses of brain tissues did not detect any infectious virus (data not shown). Examination of RNA from the lung and various CNS regions of B.1.351-infected mice via quantitative reverse transcription polymerase chain reaction (qRT-PCR) revealed elevated levels of SARS-CoV-2 subgenomic E transcript within the lungs at 2 and 4 dpi, but not in the CNS. A single animal had detectable transcript within the forebrain close to the limit of detection of the assay (Fig. 1b). B.1.351 infection significantly increased levels of antiviral cytokines, including IFN-β, IFN-γ, IL-1β, and TNF at 4 and 6 dpi in the lungs (Suppl. Fig. 1d). Visualization of Spike RNA within tissues via *in situ* hybridization (ISH) revealed widespread infection of the lungs at 4 dpi, while analysis of the entire brain did not detect Spike RNA signal at any time-point (Fig. 1c and Suppl. Fig. 1e). Together, these data show that B.1.351 infects the respiratory tract, but not the CNS, of wild type C57Bl/6J mice.

To determine if B.1.351 infection of C57Bl/6J mice led to alterations in behavior after recovery, we performed open field (OFT) and novel object recognition (NOR) testing at 30 dpi (Fig. 1d). OFT, which assesses general motor function and anxiety, revealed a small, but statistically significant, decrease in mean movement speed (Fig. 1e). However, the number of lines crossed, total rotations, and time spent immobile were not different between mock- and B.1.351-infected groups (Fig. 1e, Suppl. Fig. 1f). Similarly, the amount of time spent in corner vs center zones did not reveal any differences (Fig. 1e). NOR testing, which investigates brain networks underlying recognition memory, revealed that both mock- and B.1.351-infected animals investigated two identical objects an equal number of times on the training day^40^. On test day, mock-infected mice show a significant preference for the novel object, while B.1.351-recovered mice show no discrimination between the objects (Fig. 1f). This is reflected in a significant decrease in the discrimination index (D.I.), which measures the difference in time spent between the novel and old object, in B.1.351- compared with mock-infected mice (Fig. 1g). Preference testing confirmed there was no innate bias for either of the two objects used in the NOR test (Suppl. Fig. 1g). Analysis of OFT and NOR test data did not show any significant differences between sexes (Suppl. Fig. 1h-i). There was no correlation between weight loss and NOR test performance (Suppl. Fig. 1i). Combined, these data show that i.n. infection of C57Bl/6J with B.1.351 causes memory deficits at post-acute timepoints.

### B.1.351 infection induces CNS monocyte infiltration and microglial activation

**Figure 2:**
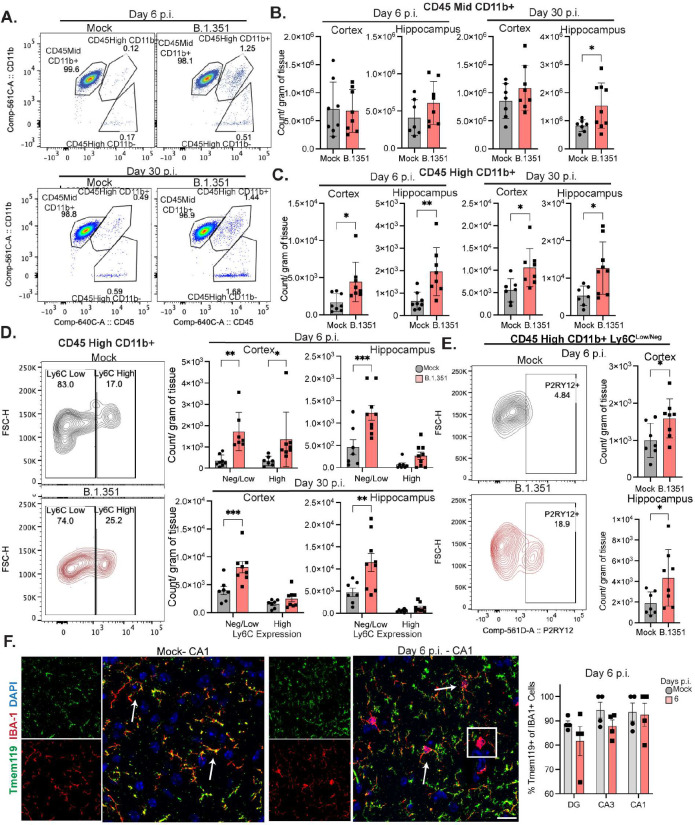
B.1.351 infection induces microglial activation and monocyte infiltration in the CNS. C57Bl/6J mice were intranasally infected with B.1.351 and at the indicated timepoint analyzed via flow cytometry. A) Representative pseudocolor plots of CD45 vs CD11b expression on myeloid cells (Singlets, Live Cells, CD45+, Ly6G−, CD3−, CD19−) showing gating strategy for CD45^mid^CD11b+, CD45^high^CD11b+, and CD45^high^CD11b− at 6 and 30 dpi. B) Quantification of number of CD45^mid^CD11b+ or C) CD45^high^CD11b+ cells per gram of tissue for the cortex and the hippocampus at 6 and 30 dpi (n=7–9). D) Representative contour plots of Ly6C expression on CD45^High^CD11b+ cells from the cortex at 6 dpi. On the right, quantification of the number of Ly6C^High^ or Ly6C^low/Negative^CD45^High^CD11b+ myeloid cells (n=7–9). E) Representative contour plots of P2RY12+CD45^High^CD11b+Ly6C^low/neg^ cells, cell number is quantified on right in cortex and hippocampus for 6 dpi (n=7–8). F) Representative z-stack of Tmem119, IBA-1 staining from mock or B.1.351 mice at 6 dpi in the CA1. On the right, frequency of IBA-1+ cells that are also Tmem119+. All image acquisition and analysis were performed blinded, all quantification is averaged from 2–3 sections per mouse. Scale bar is 20 μM. Data is represented as mean with SEM and was pooled from 2–3 individual experiments. Statistical significance was determined using a two-way ANOVA, or student’s t-test. *=p<0.05, **=p<0.01, ***=p<0.001.

CNS-infiltrating immune cells can promote memory and learning deficits via delivery of cytokines that alter the homeostatic functions of resident neural cells^30,31,41,42^. Flow cytometric assessment of blood detected increased percentages of Ly6C^high^ inflammatory monocytes and neutrophils (Ly6G+), and decreased percentages of B cells (CD19+) at 6 dpi, which all return to baseline frequencies by 30 dpi (Suppl. Fig. 2a-d). Next, we examined leukocyte infiltration into the cortex and hippocampus. Myeloid cell populations (CD45+, Ly6G−, CD3−, CD19−) were identified via CD45 and CD11b expression (Fig. 2a, Suppl. Fig. 2a). CD45^mid^CD11b+ cell numbers were similar between mock- and B.1.351-infected animals at 6 dpi in the cortex and hippocampus but were significantly increased in the hippocampus at 30 dpi (Fig. 2b). Importantly, CD45^high^CD11b+ cell numbers were significantly increased in the cortex and hippocampus at 6 and 30 dpi. Numbers of B-cells (CD19+) and T cells (CD3+) were increased at 6 dpi within the cortex, and T cells remained elevated compared to mock-infected animals at 30 dpi (Suppl. Fig. 2e-f). CD45^high^CD11b- cells, which are primarily dendritic cells or Natural Killer cells, were increased in number at 30 dpi, but not at 6 dpi, in the cortex (Suppl. Fig. 2e-f). No differences were observed in the number of neutrophils (Ly6G+) between mock- and B.1.351-infected mice (Suppl. Fig. 2e-f). Combined these data demonstrate that T cells and myeloid cells accumulate in the cortical and hippocampal parenchyma starting at acute timepoints and remain elevated at recovery timepoints.

During inflammatory conditions in the brain, myeloid cell populations can consist of microglia (CD45^mid/high^, P2RY12+, Ly6C^neg^), resident macrophages (CD45^high^, P2RY12-, Ly6C^low/neg^), or infiltrating monocytes (CD45^high^, P2RY12−, Ly6C^low/high^). After B.1.351 infection, CD45^mid^CD11b+ cells were 99% P2RY12+Ly6C^low/neg^ (microglia) (Suppl Fig. 3a). CD45^high^CD11b+ cells consisted of a Ly6C^low/neg^ (75–80%) and a Ly6C^high^ (20–25%) population, both of which increased in the cortex and hippocampus of B.1.351-infected mice at 6 dpi. However, at 30 dpi, Ly6C^high^ cell numbers were the same as in mock-infected mice, while the number of Ly6C^low/neg^ cells was still higher in B.1.351-infected animals (Fig. 2d). Further analysis of the CD45^High^CD11b+ Ly6C^low/neg^ population showed that at 6 dpi, ~20% of the cells were P2RY12+ and significantly increased in number compared to mock-infected animals (Fig. 2e). To examine myeloid cell location within the hippocampus, we performed immunohistochemical (IHC) detection of the myeloid activation marker IBA-1. In all regions of the hippocampus, IBA-1+ area significantly increased in B.1.351-infected mice at 6 dpi compared to mock-infected animals (Suppl. Fig. 3b). IHC detection of IBA-1 and the microglial marker Tmem119 in hippocampi from mock-infected mice revealed that 90% of IBA-1+ cells were also Tmem119+, compared with 80–90% of IBA-1+ cells of hippocampi from B.1.351-infected mice at 6 dpi. Few cells were IBA-1+Tmem119− in our analyses (Fig. 2f). These data indicate that a small, but significant number of Ly6C^high^ inflammatory monocytes infiltrate the forebrain at acute timepoints and contract over time, while microglial activation persists long-term.

### Activated myeloid cells produce IL-1β during acute B.1.351 infection.

**Figure 3:**
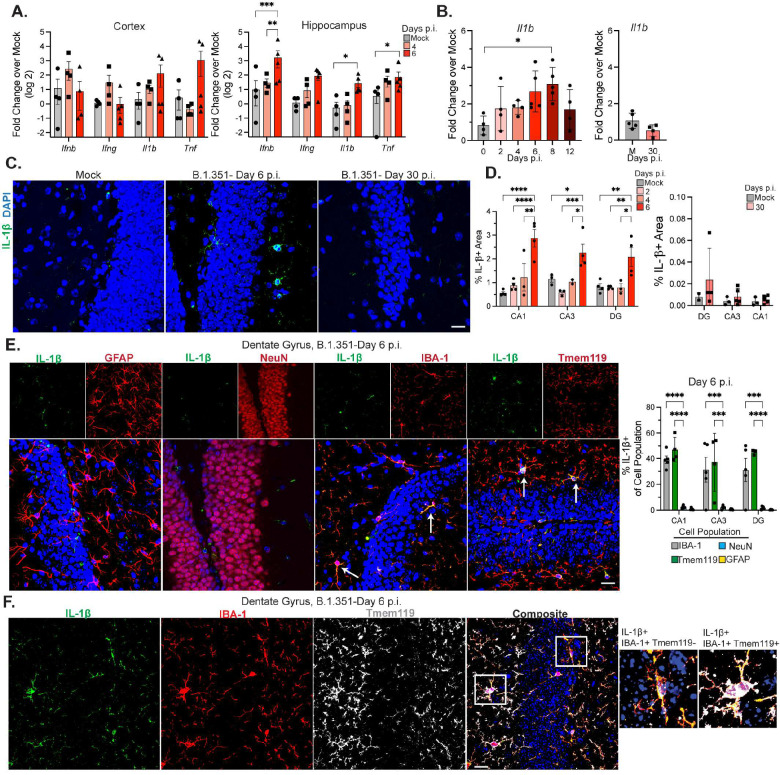
Activated myeloid cells produce IL-1β during acute B.1.351 infection. Mice were intranasally infected with B.1.351. A) Transcript levels of indicated gene as compared to mock (via qRT-PCR) at 4 and 6 dpi in the cortex and hippocampus (n=3–4). B) Transcript levels of *Il1b* from 0–12 dpi and at 30 dpi represented as fold-change over respective mock (n=3–5). C) Representative z-stacks of IL-1β staining in the DG at 6 and 30 dpi. Mock is from 6 dpi. D) Quantification of the percent IL-1β+ area at 2, 4, 6 dpi (left) or 30 dpi (right) with the respective mocks (6 and 30 dpi) in the DG, CA3, and CA1. E) Representative z-stacks of IL-1β co-stained with one of the following: GFAP, NeuN, IBA-1, or Tmem119 at 6 dpi in the DG. Arrows indicate cell marker stains that co-localize with IL-1β signal. On the right, the Manders co-efficient for each cell type expressed as percentage of IL-1β+ staining that co-localizes with the indicated cellular marker (n=4–5). F) Representative z-stacks demonstrating co-localization of IBA-1, Tmem119, and IL-1β at 6 dpi in the DG. White boxes highlight zoomed in images on right. All image acquisition and analysis was performed blinded, all quantification is averaged from 2–3 individual sections for each mouse. Scale bar is 20 μM. Data is represented as mean with SEM and is representative of 2–3 individual experiments. Statistical significance was determined using a one-way or two-way ANOVA, or student’s t-test. *=p<0.05, **=p<0.01, ***=p<0.001.

Given the CNS infiltration of inflammatory monocytes observed within acutely infected B.1.351 animals, we examined cytokine expression via qRT-PCR in forebrain tissues from B.1.351-infected mice at 4 and 6 dpi. B.1.351 significantly increased hippocampal levels of IFN-β, IL-1β, and TNF at 6 dpi. While cytokine levels were increased in the cortex, this did not reach significance (Fig. 3a). To determine if this was a generalizable effect of severe respiratory infections, or specific to SARS-CoV-2, we i.n. infected mice with a high dose of the mouse adapted H1N1 influenza A virus (IAV) (strain A/Puerto Rico/8/1934; 2000 TCID_50_). Despite up to 20% loss of body weight, PR8 does not infect the CNS (Suppl. Fig. 4a-b). PR8 infection induced cytokine expression in the lung, but no significant differences in IL-1β, IL-1α, IFNγ, or IFNβ mRNAs were detectable in the forebrain at 3 or 6 dpi compared with mock-infected animals (Suppl. Fig. 4c). Due to the known anti-neurogenic effects of IL-1β, we examined its expression within the hippocampus throughout B.1.351 infection^30^. IL-1β mRNA levels were significantly elevated in SARS-CoV-2- versus mock-infected animals, peaking at 6–8 dpi then declining by 12 dpi and returning to mock levels at 30 dpi (Fig. 3b). Consistently, IHC of hippocampi from B.1.351-infected mice at 6 dpi exhibited significantly increased levels of IL-1β compared to mock animals, which became undetectable at 30 dpi (Fig. 3c–d, Suppl Fig. 4d). IL-1β mRNA and protein were undetectable in all CNS tissues derived from H1N1-infected mice (Suppl. Fig. 4c,e). In B.1.351-infected mice, IL-1β was not detected within GFAP+ or NeuN+ cells (Fig. 3e). However, approximately 80–90% of IL-1β within hippocampi at 6 dpi was detected within IBA-1+ and Tmem119+ cells (Fig. 3e–f). Despite the lack of IL-1β expression, there was a significant increase in the percentage of IBA-1+ area after H1N1 IAV infection in the DG and CA3 (Suppl. Fig. 4f). Together, these data indicate that infiltrating monocytes and microglia transiently increase IL-1β levels in the hippocampus after i.n. B.1.351 infection.

### Hippocampal neurogenesis and synapses decrease after B.1.351 infection.

**Figure 4:**
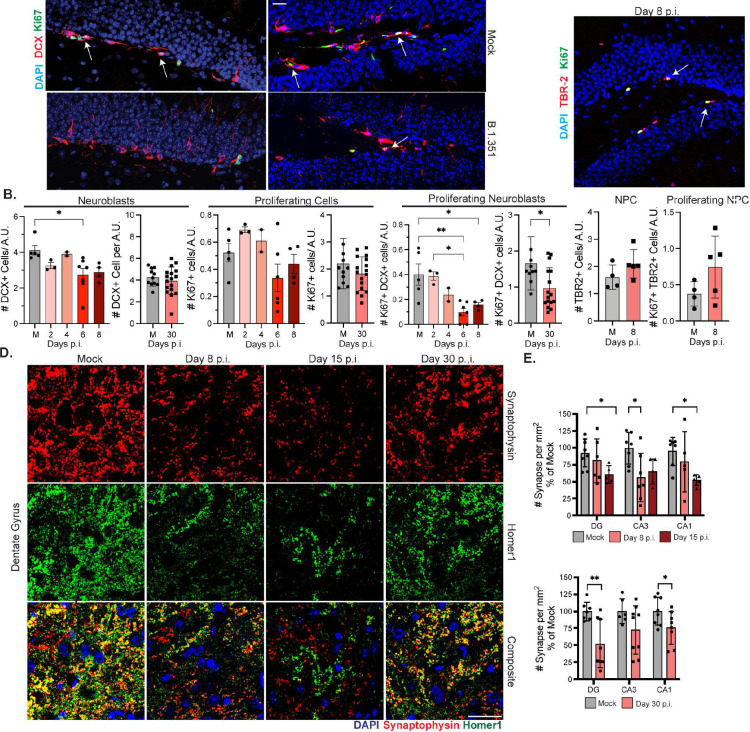
Hippocampal neuroblast proliferation and synapse number decrease after B.1.351 infection. Mice were intranasally infected with B.1.351. A) Representative images of Ki67 and doublecortin (DCX) staining in the SGZ of the DG at 6 and 30 dpi, with their respective mocks. Arrows point to co-localization of Ki67 and DCX. B) Number of DCX+, Ki67+, or DCX+Ki67+ cells counted along the SGZ and divided by the length of the DG expressed as Arbitrary Units (A.U.). Samples from 0–8 dpi are compared to a mock (M) from 6 dpi (n=3–6), mice from 30 dpi are compared to a mock from 30 dpi (n=10–15). C) Representative image of TBR2 and Ki67 staining in the DG at 8 dpi. Quantified below is the number of TBR2+ or TBR2+Ki67+ cells per DG length in A.U. compared to 8 dpi mock (M, n=4–5). D) Representative z-stacks of synaptophysin and homer-1 staining in the DG of mice at 8, 15, or 30 dpi. Yellow signal indicates co-localized synapse terminals. E) Quantification of number of overlapping synaptic terminals expressed as a percentage of the average number of mock synapses for each region surveyed (DG, CA3, CA1). 8 and 15 dpi are compared to 8 dpi mock (n=5–6). 30 dpi is compared to a day 30 mock (n=7–8). All image acquisition and analysis were performed blinded and quantification was averaged from 3–5 individual sections. For synapse quantification 2 images per region were taken for each section. Scale bar is 20 μM. Data is represented as mean with SEM and pooled from or representative of 2–3 independent experiments. Statistical significance was determined using a one-way or two-way ANOVA, or student’s t-test. *=p<0.05, **=p<0.01, ***=p<0.001.

Given the memory deficits observed in SARS-CoV-2-recovered mice and the elevated levels of IL-b in the hippocampi of B.1.351-infected mice, we evaluated adult hippocampal neurogenesis and synapses^28–31^. B.1.351 infection significantly decreased the total number of doublecortin (DCX)+ neuroblasts at 6–8 dpi, which recovered by 30 dpi. The number of proliferating neuroblasts (DCX+ Ki67+) was significantly decreased compared with mock animals from 6–8 dpi and remained lower at 30 dpi (Fig. 4a–b). Evaluation of Type 2 intermediate neuronal progenitors (NPC) via IHC detection of T-box protein (TBR2/EOMES) revealed no changes in numbers of total TBR2+ or proliferating (TBR2+Ki67+) NPCs (Fig. 4c). H1N1 infection did not impact neuroblasts or proliferating neuroblasts at 6 dpi (Suppl. Fig. 5a). Thus, SARS-CoV-2 induces loss of adult neurogenesis via inhibition of DCX+ neuroblast proliferation.

Next, we quantitated synaptic puncta within the DG, CA3 and CA1 regions via co-localization of the pre-synaptic marker, Synaptophysin, and the post-synaptic marker, Homer1. Synapse loss was observed throughout the hippocampus, beginning at 8 dpi and was significantly decreased by 15 dpi (Fig. 4d–e). Synapses partially recovered in the CA3 by 30 dpi but remained decreased in the CA1 and DG compared to mock-infected mice (Fig. 4e). Analysis of individual pre-synaptic/post-synaptic termini indicate that in the DG, synapse loss is primarily driven by decreased pre-synaptic termini, while in the CA3, post-synaptic termini are lost (Suppl. Fig. 5b-c). TUNEL staining for apoptotic cells confirmed that synapse loss was not due to excessive neuronal death, as TUNEL+ NeuN+ numbers were extremely low and equal between mock- and B.1.351-infected mice (Suppl. Fig. 5d). These data identify acute and post-acute loss of synapses in the hippocampus after COVID-19.

### IL-1R1 signaling on neural stem cells mediates acute loss of neurogenesis and memory deficits after B.1.351 infection.

**Figure 5:**
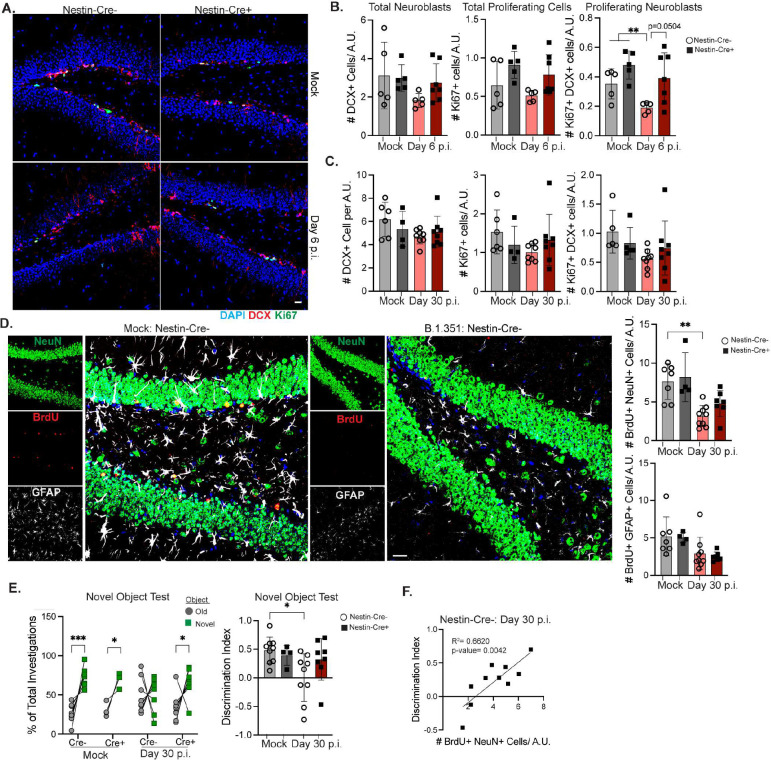
IL-1R1 signaling on NSCs promotes acute loss of neurogenesis and memory deficits. Nestin-Cre^ERT2^ x IL-1R1^fl/fl^ littermates (Cre+ and Cre−) were intraperitoneally (I.P.) injected with Tamoxifen for 5 days. 10 days after the last tamoxifen injection, mice were intranasally infected with B.1.351. A) Representative images of Nestin-Cre− or Nestin-Cre+ littermates from 6 dpi mock or B.1.351 infected animals showing Ki67, DCX staining in the DG. Quantification of the number of DCX+, Ki67+, or DCX+Ki67+ cells per A.U. of DG length between Cre+ and Cre− Mock or B.1.351 littermates at B) 6 dpi (n=5–7) C) or 30 dpi (n=4–7). D) At 5–7 dpi, Cre+ and Cre− littermates were given BrdU I.P. every 12 hours. Representative Z-stack from Cre− mock or B.1.351 animals at 30 dpi of BrdU, NeuN, GFAP in the DG. To the right, number of BrdU+NeuN+ or BrdU+GFAP+ cells per A.U. of DG length (n=3–7). E) The percentage of investigations (nose poke) of old vs novel object measured during the NOR test for mock and B.1.351 infected Nestin-Cre+ and Cre− littermates at 30 dpi. Individual mice are connected with a line. On the right, the discrimination index is quantified for each mouse (n=4–10). F) Linear regression analysis comparing the correlation between the Discrimination Index and the number of BrdU+NeuN+ cells for Cre− B.1.351 infected mice at 30 dp (n=10). All image analysis and acquisition were performed blinded and quantification was averaged from 3–5 slices for each mouse. Scale bar is 20 μM. Data is represented as mean with SEM and was pooled from 2–4 independent experiments. Statistical significance was determined using simple linear regression, one-way ANOVA, two-way ANOVA, student’s t-test, or paired two-way ANOVA (for F). *=p<0.05, **=p<0.01, ***=p<0.001.

In prior studies, we identified neural stem cells (NSC) as the target of IL-1β mediated loss of neurogenesis during neurotropic viral infection^30^. To determine if this underlies SARS-CoV-2-mediated loss of neurogenesis and memory deficits, we utilized a Nestin-Cre^ERT2^ × *Il1r1*^fl/fl^ mouse model, in which IL-1R1 is deleted from NSCs after tamoxifen injection^30^. Cre+ and Cre− littermates were intraperitoneally injected with tamoxifen for 5 days; 10 days later, mice were infected with B.1.351, which produced no differences in weight loss between Cre+ and Cre− mice (Suppl Fig. 6a). At 6 dpi, B.1.351 infected Cre− mice had significantly reduced numbers of proliferating neuroblasts (Ki67+ DCX+) compared to mock-infected and trended decreased compared to B.1.351-infected, Cre+ mice (Fig. 5a–b). However, at 30 dpi no statistical differences in proliferating neuroblasts were found between all groups, although proliferating neuroblasts trended downwards in B.1.351 Cre− mice compared to mock Cre− controls (Fig. 5c, Suppl. Fig. 6b). These data indicate that IL-1R1 signaling promotes the acute loss of neuroblast proliferation during SARS-CoV-2 infection.

To determine whether loss of adult neurogenesis during COVID-19 is associated with increased astrogenesis, as observed in neuroinvasive models of viral infection, we utilized BrdU-incorporation^30^. Tamoxifen-treated, Cre− and Cre+ mice were intraperitoneally injected with BrdU (50 mg/kg) every 12 hours from 5 to 7 dpi. At 30 dpi, BrdU+ astrocytes (GFAP+) or BrdU+ neurons (NeuN+) were quantified within the DG (Fig. 5d). The numbers of newly generated neurons were significantly decreased in B.1.351- compared to mock-infected Cre− and Cre+ mice, although the trend was not significant for Cre+ B.1.351 mice. However, there were no differences in the numbers of newly generated astrocytes (BrdU+ GFAP+) after infection or between Cre− and Cre+ mice (Fig. 5d). These data show that B.1.351 infection does not impact astrogenesis but leads to a decrease in newly generated neuron numbers even in the absence of IL-1R1 expression on NSCs.

Quantitation of hippocampal synapses via co-localization of Synaptophysin and Homer1 in Nestin-Cre^ERT2^ x IL-1R1^fl/fl^ mice did not demonstrate a role for IL-1R1 in the loss of synapses or pre-synaptic/ post-synaptic termini after B.1.351 infection (Suppl. Fig. 6c-d). However, NOR testing of mock- versus SARS-CoV-2-recovered Cre− and Cre+ mice revealed a critical role for NSC IL-1R1 in this cognitive task. As expected, on the training day, mice showed no preference for the two identical objects (Suppl. Fig. 6e) and on the NOR test day, mock Cre− and Cre+ mice show a preference for the novel object (D.I.=0.5). B.1.351-infected Cre− mice show no preference for the novel object (D.I=0.0). In contrast, B.1.351-infected Cre+ mice show a significant preference for the novel object (D.I.=0.3, Fig. 5e). OFT did not show any differences in motor activity; however, we did observe a significant increase in time spent in the center zone and a decrease in time spent in the corner zones between Cre− mock-infected and Cre− / Cre+ B.1.351-infected mice, respectively (Suppl. Fig. 6f). Correlation analyses found a significant positive correlation between numbers of newly generated neurons (BrdU+ NeuN+) and higher discrimination indexes in Cre− mice (Fig. 5f). No significant correlations were observed between the D.I. and the number of recently proliferated astrocytes (BrdU+ GFAP+) or neuroblasts (Ki67+ DCX+). There was a weak positive correlation between synapse number and D.I. score (Suppl. Fig. 6g). Combined, these data find that IL-1R1 signaling on NSCs decreases neurogenesis during acute B.1.351 infection and the resulting loss of newly generated neurons in the DG promotes post-acute memory deficits.

### Vaccination reduces IL-1β within the hippocampus during breakthrough SARS-CoV-2 infection

**Figure 6:**
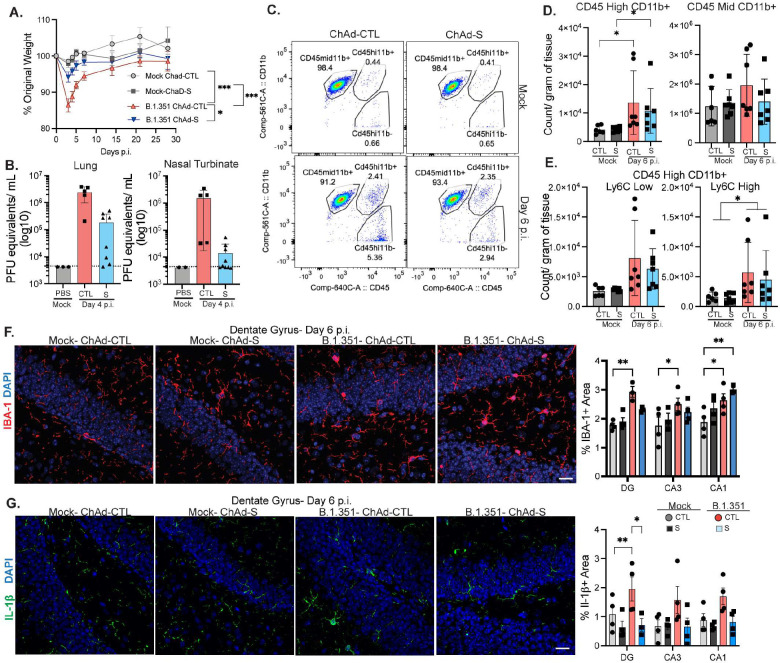
Vaccination lowers hippocampal IL-1β expression after breakthrough infection. Mice were intranasally vaccinated with 10^8^ ChAd-S or an empty vector control (ChAd-CTL). 30 days later, mice were challenged with intranasal B.1.351. A) Percentage of original weight after infection with B.1.351 or mock in ChAd-S and ChAd-CTL animals (n=10). B) Viral titer measured via plaque assay in the lung and nasal turbinates at 4 dpi from B.1.351 infected ChAd-S and ChAd-CTL animals. Dotted line is limit of detection (n=5–8). C) Representative pseudocolor plots showing CD45 vs CD11b expression on myeloid cells (CD45+, Ly6G−, CD19−, CD3−) from the forebrain. D) Number of CD45High CD11b+ or CD45Mid CD11b+ cells per gram of tissue from the forebrain at 6 dpi. E) Number of Ly6C^Low^ or Ly6C^High^CD45^High^CD11b+ cells (n=8). F) Representative images of IBA-1 at 6 dpi from the DG. On the right, percentage of IBA-1+ area in the indicated region (n=4–5). G) Representative images of IL-1β at 6 dpi from the DG. On the right, percentage of IL-1β+ area in the indicated region (n=4–5). All image analysis and acquisition were performed blinded and quantification was averaged from 2–4 slices for each mouse. Scale bar is 20 μM. Data is represented as mean with SEM and was pooled or representative of 2 independent experiments. Statistical significance was determined using simple linear regression, one-way ANOVA, two-way ANOVA, student’s t-test, or paired two-way ANOVA (for F). *=p<0.05, **=p<0.01, ***=p<0.001.

In patients, vaccination against SARS-CoV-2 may decrease the risk of developing neuroPASC after a breakthrough SARS-CoV-2 infection^43,44^. To determine whether vaccination alters CNS levels of IL-1β and neural correlates of learning, we developed a model of breakthrough infection after vaccination of C57Bl/6J mice with a chimpanzee-adenoviral vector (ChAd) vaccine containing the pre-fusion Spike protein of the original Wuhan virus, which has been shown to protect against pneumonia in rodents^46^. Mice i.n. vaccinated with 10^8^ ChAd-Spike (ChAd-S) (Suppl. Fig. 7a) developed low levels of neutralizing antibodies to B.1.351 at 21 days post-vaccination, while animals administered ChAd-Empty Vector Control (ChAd-CTL) did not (Suppl. Fig. 7b). At day 30 post-vaccination, mice were challenged i.n. with B.1.351 (5 × 10^5^ pfu). ChAd-CTL mice lost ~15% of their total body weight at 4 dpi., while ChAd-S mice lost only 5% of their body weight (Fig. 6a). Mice vaccinated with ChAd-S had detectable B.1.351 virus in the lung and nasal turbinate, however, compared to ChAd-CTL mice, viral load was decreased 10-fold and 100-fold respectively (Fig. 6b). Thus, we developed a vaccination model that provides incomplete protection against challenge and allows breakthrough infection with B.1.351.

Examination of immune cell numbers at 6 dpi within the forebrain revealed that CD3+ cells were significantly increased after B.1.351 infection in ChAd-CTL but not in ChAd-S mice (Suppl. Fig. 7c). CD45^high^CD11b+ cells were significantly increased in number at 6 dpi in ChAd-CTL and ChAd-S mice (Fig. 6c–d). Numbers of CNS CD45^mid^CD11b+ cells were unchanged by vaccination. B.1.351 infection increased numbers of CD45^high^CD11b+Ly6C^low^ and CD45^high^CD11b+Ly6C^high^ cells compared to mock animals, regardless of vaccination status (Fig. 6e). IHC detection of IBA-1 within the hippocampi of ChAd-CTL vaccinated mice at 6 dpi revealed significantly higher levels of IBA-1 compared to mock controls in every region, while only the CA1 region exhibited this in ChAd-S, B.1.351-infected mice (Fig. 6f). Finally, IHC detection of IL-1β expression in the hippocampi of vaccinated animals revealed that ChAd-CTL, but not ChAd-S, B.1.351-infected mice exhibited significantly higher levels of IL-1β compared to mock-infected animals at 6 dpi (Fig. 6g). Combined, these data indicate that vaccination with ChAd-S prevents expression of hippocampal IL-1β.

### Vaccination prevents loss of adult neurogenesis and cognitive impairments after recovery from breakthrough SARS-CoV-2 infection.

**Figure 7:**
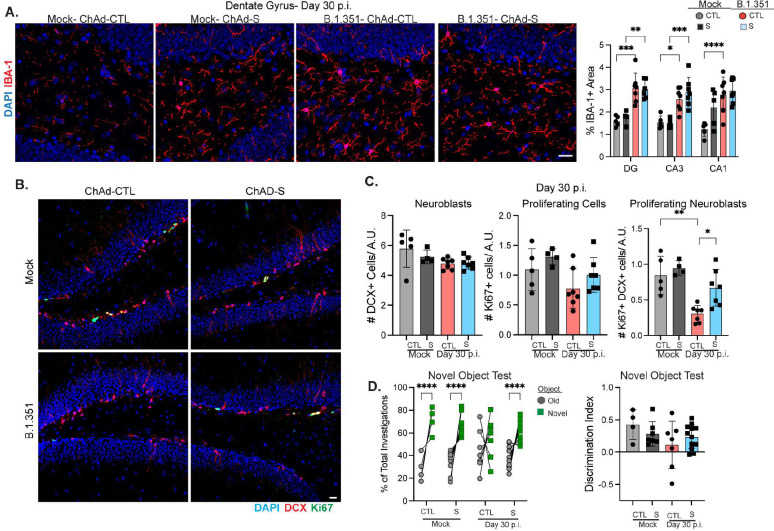
Vaccination prevents loss of neurogenesis and cognitive deficits after breakthrough SARS-CoV-2 infection. Mice were intranasally vaccinated with ChAd-S or an empty vector control (ChAd-CTL). 30 days later, mice were challenged with intranasal B.1.351. A) Representative images of IBA-1 expression in the DG of ChAd-S or ChAd-CTL (mock or B.1.351 infected) at 30 dpi. On the right, percentage of IBA-1+ area in the indicated hippocampal region at 30 dpi (n=5–8). B) Representative images of Ki67, DCX staining in the SGZ of the DG at 30 dpi from mock and B.1.351 infected ChAd-S or ChAd-CTL mice. C) Number of DCX+, Ki67+, or DCX+Ki67+ cells/ DG length (in A.U.) at 30 dpi (n=4–8). D) The percentage of the total investigations spent with the Old or Novel object during NOR testing at 30 dpi. Individual animals are connected with a line. On the right, the discrimination index (n=4–10). All image analysis and acquisition were performed blinded and quantification was averaged from 2–4 slices for each mouse. Scale bar is 20 μM. Data is represented as mean with SEM and was pooled from 2 independent experiments. Statistical significance was determined using, one-way ANOVA, two-way ANOVA, student’s t-test, or paired two-way ANOVA (for F). *=p<0.05, **=p<0.01, ***=p<0.001, ****=p<0.0001.

Given that vaccination prevents hippocampal IL-1β expression, we investigated whether it would also rescue alterations in adult neurogenesis and post-acute cognitive deficits in B.1.351-recovered mice. At 30 dpi, analysis of IBA-1 expression in the hippocampi of ChAd-CTL and ChAd-S animals revealed significantly increased levels of IBA-1 in every region surveyed regardless of vaccination status (Fig. 7a), indicating that microgliosis occurs during breakthrough infections despite lower levels of respiratory virus and less acute neuropathology. Numbers of neuroblasts (DCX+) or total proliferating cells (Ki67+) did not differ between mock- and B.1.351-recovered mice regardless of vaccination. However, proliferating neuroblast (Ki67+DCX+) cell numbers were significantly decreased in ChAd-CTL, but not in ChAd-S, B.1.351-infected mice compared with mock controls (Fig. 7b–c). Behavioral testing revealed no differences in OFT or NOR training day performance (Suppl. Fig. 8a-b). Mock-infected, ChAd-S vaccinated mice performed similarly to mock ChAd-CTL vaccinated mice on the NOR test, spending ~ 70% of their time with the novel object (D.I.= ~0.4). As expected, B.1.351 infected ChAd-CTL mice had no preference for the novel object (D.I.=0.1). In contrast, ChAd-S, B.1.351 infected mice showed a significant preference for the novel object, spending ~60% of their investigations with the novel object (D.I.=0.3, Fig. 7d). Together these data indicate that i.n. vaccination with ChAd-S prevents loss of neurogenesis and memory deficits after recovery from breakthrough B.1.351 infection in mice.

## Discussion

In this study, we developed a mouse model of neuroCOVID using intranasal B.1.351 infection of C57Bl/6J mice and a model of breakthrough infection after vaccination against SARS-CoV-2 Spike protein. B.1.351 induces robust infection and inflammation of the respiratory tract, but also induces Ly6C^high^ monocyte infiltration and elevated pro-inflammatory cytokines in the forebrain of infected mice at acute timepoints^37,39^. IL-1β produced by activated microglia acts on NSCs to inhibit neuroblast proliferation within the SGZ during acute infection. Decreased neurogenesis persists at 30 dpi and along with loss of hippocampal synapses drives cognitive deficits. Importantly, elevated hippocampal IL-1β and decreased neurogenesis are not observed during H1N1 infection of mice. Furthermore, vaccination against S protein reduces acute hippocampal IL-1β expression during breakthrough infection, with associated rescue of neurogenesis and cognitive ability at 30 dpi. Together, these studies identify IL-1β as a key driver of hippocampal dysfunction during neuroPASC and indicate that vaccination limits neuroinflammation during breakthrough infection.

In our study, we found that i.n. infection of C57Bl6/J mice with B.1.351 produces post-acute cognitive deficits. Incidence of neuroCOVID in humans ranges from 15–60%, and in our mouse model we also observe variability, with approximately 50% of mice performing poorly on the NOR test^3–7^. NeuroCOVID risk in humans is increased with hospitalization and in females, however NOR test performance in mice did not correlate with disease severity or with biological sex^4,5,7^. At 30 dpi, the number of proliferating neuroblasts and hippocampal synapses were also highly variable in B.1.351 infected mice and this directly correlated to NOR test performance, despite uniform loss at acute timepoints. SARS-CoV-2-recovered mice also exhibited a slight decrease in movement speed, which could suggest either fatigue or motor deficits. Due to the constraints of performing behavioral tests in a BSL-3 level biosafety cabinet we were unable to perform additional motor function tests. Thus, B.1.351 infection of C57Bl/6J mice can be used to investigate COVID-19 memory dysfunction, but may not be relevant for other symptoms, such as anxiety, which we did not observe in our model.

B.1.351 infection of mice was associated with significant infiltration of inflammatory monocytes into the forebrain. However, monocyte infiltration has not consistently been observed in post-mortem brains from COVID-19 patients^47,48,49^. This could be explained by our finding that Ly6C^high^ monocytes infiltrate the brain for a brief period during acute infection, and samples from such early time-windows in humans are rare. Inflammatory monocytes derived from COVID-19 patients exhibit high levels of inflammasome activation and IL-1β expression^50,51^. Similarly, we observed increased IL-1β within the forebrain of B.1.351 infected mice, which localized to activated macrophages and microglia within the hippocampus. Microgliosis and IL-1β expression within myeloid cells of the CNS have been reported in COVID-19 patients and in SARS-CoV-2 infected hamsters^18,52^. While inflammatory monocytes are no longer found in the hippocampus at 30 dpi, activated microglia do persist, similar to findings in the human COVID-19 brain^18,20,23,23,47,49,52^. Non-microglial P2RY12-, CD45High, CD11b+, Ly6C^Low^ cells are also detected, which could be CNS-resident macrophages or infiltrating monocytes that have downregulated Ly6C. Our study also confirms previous work that demonstrates increased numbers of T cells in the brains of patients with COVID-19^20,47,49^. In our murine model, IFNγ mRNA levels trend increased at 6 dpi and T cell numbers are elevated at acute and post-acute timepoints. IFNγ promotes microglial-mediated synapse engulfment that contributes to cognitive deficits^53,54^. Future studies are needed to help define the contribution of persistently activated microglia and T cells to cognitive deficits during neuroPASC.

While this study provides the first demonstration of a respiratory virus causing IL-1β-mediated inhibition of hippocampal neurogenesis, this has been previously described during infection of mice with neuroinvasive West Nile virus (WNV)^30,31^. However, in contrast with SARS-CoV-2 infection, deletion of IL-1R1 from NSCs not only rescues loss of hippocampal neurogenesis during WNV encephalitis, but also leads to recovery of hippocampal synapses^30,31^. In addition, during WNV infection of the CNS, IL-1R1 signaling in NSCs promotes astrogenesis at the expense of neurogenesis, which was not observed in our murine COVID-19 model, potentially because the block in neurogenesis did not occur until the neuroblast stage of differentiation, after commitment to a neuronal cell fate. Additionally, newly generated astrocytes become a new source of IL-1β during WNV recovery, whereas IL-1β levels fall in SARS-CoV-2-recovered mice and deletion of NSC IL-1R1 was not able to fully rescue new neuron generation at 30 dpi^30,31^. Thus, IL-1β is not involved in post-acute loss of neurogenesis and synapses after COVID-19. Further work is needed to define the mechanisms underlying persistent inhibition of neurogenesis and memory deficits after recovery from COVID-19.

Peripheral inflammation, including high serum levels of IL-1β, has been linked to hippocampal dysfunction in other disease models^55^. Severe COVID-19 is associated with high levels of IL-1β, even compared to other respiratory infections, such as influenza^56^. In our study, comparison with H1N1 infection revealed that IL-1β expression in the hippocampus is a unique feature of SARS-CoV-2, although, in agreement with previous work, H1N1 does cause some hippocampal immune activation^52,57–59^. Furthermore, we demonstrate that vaccination alone has no impact on hippocampal function and even low dose, strain-mismatched vaccination prevents IL-1β production, loss of neurogenesis, and memory deficits after breakthrough SARS-CoV-2 infection. These data agree with human studies that vaccination is associated with reduced peripheral inflammation and risk of long-COVID, and demonstrate that one mechanism by which cognitive deficits may be prevented is through inhibition of hippocampal IL-1β expression^33–35^. Interestingly, we find that hippocampal myeloid cell activation does not always lead to IL-1β production, as activated microglia/macrophages were observed in the hippocampus of H1N1 infected or B.1.351-infected, vaccinated mice, but did not produce IL-1β. This suggests that SARS-CoV-2 infection produces a unique stimulus not found in H1N1 or after vaccination.

In summary, we developed a mouse model that recapitulates cognitive deficits during neuroPASC. We utilized this model to discover a critical period during acute SARS-CoV-2 infection, where activated myeloid cells produce IL-1β in the forebrain, which acts as a key driver of hippocampal dysfunction during COVID-19. We then demonstrate that vaccination limits IL-1β mediated loss of neurogenesis and cognitive deficits during breakthrough SARS-CoV-2 infection. Thus, IL-1β signaling is a potential therapeutic target for individuals suffering from memory deficits post-COVID-19.

## Methods

### Viruses and cells

VeroE6-hACE-2-TMPRSS2 or VeroE6-TMPRSS2 cells were a generous gift from Dr Michael S. Diamond at Washington University in St. Louis and cultured in complete Dubecco’s modified Eagle’s Medium (DMEM) supplemented with 10% fetal bovine Serum (FBS), 25 mM HEPES buffer, 1 mM Sodium pyruvate, and 1X antibiotics. Sequence confirmed SARS-CoV-2 B.1.351 (Beta variant) was a generous gift from Dr. Mehul Suthar at Emory University in Atlanta^37^. All B.1.351 stocks were grown on Vero-E6-TMPRSS2 cells and viral titers were determined by plaque assays on Vero-E6-hACE-2-TMPRSS2 cells. WNV-NS5-E218A, which harbors a single point mutation in the 2′ *O*-methyl-transferase gene, was obtained from Dr. Michael Diamond (Washington University) and passaged in Vero cells as described previously^60^. H1N1 PR8 virus was produced as previously described^61^. A recombinant (or reverse genetics derived) H1N1 influenza A virus (IAV, strain A/Puerto Rico/8/1934) was expanded in 10-day old embryonated chicken eggs, aliquoted and stored at −80°C. The infectious virus titer was determined on MDCK cells by TCID_50_ assay^61^.

### Animals

All mouse experiments adhered to the guidelines approved by the Washington University in St. Louis Institutional Animal Care and Use Committee. C57Bl/6J mice were purchased from Jackson laboratories. Nestin-Cre^ERT2^x Il1r1^fl/fl^ mice were bred in house at Washington University in St. Louis, and verification of Cre specificity was previously described in Soung et al., 2022^30^. All mice were between 14–16 weeks of age, unless otherwise specified. Both male and female mice were used, and consideration of sex as a biological variable is demonstrated in Supplemental Figure 1. For all Nestin-Cre^ERT2^x Il1r1^fl/fl^ experiments, Cre+ animals are compared to Cre− littermates.

### Infections

Stock B.1.351 virus was diluted in phosphate-buffered saline (PBS) to a working concentration of 1.25 × 10^7^ PFU/mL. Mice were anesthetized with ketamine and infected intranasally with virus or PBS (40 μL per mouse, or 5 × 10^5^ PFU/mouse). Mice were monitored daily for weight loss until recovered to original weight (~ 7 days p.i.), at which point mice were monitored weekly. For PR8 infections, mice were anesthetized with isoflurane and inoculated intranasally with PR8 (diluted in PBS) or PBS (2000 TCID_50_). Mice were monitored daily for weight loss. For WNV infections, mice were anesthetized and inoculated with 1 × 10^4^ PFU of WNV-E218A via intracranial injection into the third ventricule of the brain with a 29-guage needle. Mice were monitored daily for weight loss.

### Quantification of virus

At the indicated time post infection, mice were euthanized via ketamine and perfused with ice-cold PBS. The indicated tissues were collected in 2 mL tubes filled with ceramic beads and 1% FBS-PBS. Tissue was weighed and homogenized in a Roche MagNa Lyser. Plaque assays were performed as previously described^62^. Briefly, 10-fold dilutions of tissue supernatant were overlaid on Vero-E6-hACE-2-TMPRSS2 cell monolayers and adsorbed for 1 hour. After adsorption, methylcellulose was overlaid and the cell cultures were incubated for 48 hours at 37°C. Methylcellulose was removed, and plates were fixed with 4% paraformaldehyde (PFA) in PBS for 30 minutes. Plaques were visualized using crystal violet in methanol. *In situ* hybridization for SARS-CoV-2 Spike RNA was performed as previously described using the RNAscope Probe V-nCoV2019-S (ACD, #848561)^18,37^.

### Quantitative reverse transcription-PCR

At the indicated day post infection, mice were euthanized via ketamine and perfused with ice-cold PBS. The indicated tissues were collected in 2 mL tubes filled with ceramic beads and TRIzol Reagent (Thermo Fisher, #15596026). Tissue was homogenized, and RNA was extracted from the supernatant using the Zymo DirectZol-RNA Miniprep kit (Zymo, #R2052) as per the manufacturer’s instructions. Conversion to cDNA was performed using a High Capacity reverse transcriptase cDNA kit (Thermo Fisher, # 4368813). Viral RNA was quantified using the IDT Prime Time gene expression master mix (#1055772) and Taqman gene expression primer/probe sets (see below). Cytokine RNA was quantified using Power SYBR green master mix (Thermo Fisher, #4367659) and custom IDT primers. All qPCRs were performed in 384-well plates. Unless otherwise specified, all data are reported as 2^ddC_T_. Primer sets are: Taqman Primers: SARS-CoV-2-E subgenomic-Forward (CGATCTCTTGTAGATCTGTTCTC), SARS-CoV-2-E Reverse (ATATTGCAGCAGTACGCACACA), SARS-CoV-2E-probe (FAM-ACACTAGCCATCCTTACTGCGCTTCGBBQ). PR8 Flu-A M Forward (CTTCTAACCGAGGTCGAAACGTA), PR8 Flu-A M Reverse (GGTGACAGGATTGGTCTTGTCTTTA), PR8 Flu-A M-probe (5’-FAM/TCAGGCCCCCTCAAAGCCGAG /3’-ZEN/IBFQ).SYBR primers: GAPDH-F (GGC AAA TTC AAC GGC ACA GT), GAPDH-R (AGA TGG TGA TGG GCT TCC C), Ifng-F (AAC GCT ACA CAC TGC ATC TTG G), IFNg-R (GCC GTG GCA GTA ACA GCC). IL1β-F (ACC TGT CCT GTG TAA TGA AAG ACG), Il1b-R (TGG GTA TTG CTT GGG ATC CA), Ifnb-F (CTG GAG CAG CTG AAT GGA AAG), Ifnb-R (CTT CTC CGT CAT CTC CAT AGG G), Tnfa-F (GCA CAG AAA GCA TGA TCC G), Tnfa-R (GCC CCC CAT CTT TTG GG)

### Behavior

All behavior was performed in a custom-built 40 × 40 × 40 cm open field box constructed with matte-white, non-adsorbent plastic and performed in an ABSL-3 level Biosafety Cabinet. Before starting behavior experiments, mice were allowed to acclimate in their home cage to the Biosafety cabinet for a minimum of 15 minutes. To perform Open Field testing, mice were placed in the center of the open field box and allowed to explore for 5 minutes. One day after Open Field testing, which also served to acclimate mice to the apparatus, mice underwent the training day for the Novel Object test. Mice were placed in the open field box, with two identical objects placed equidistant from the corner of the box and allowed to explore for 6 minutes. On day 3 (Novel Object Test Day), mice were returned to the Open Field apparatus, which now had one of the objects from Training Day and one novel object in the same locations as the training day. Mice were allowed to explore for 6 minutes. In between each mouse, the Open Field box and any test objects were thoroughly cleaned with 70% Ethanol. After testing, mice were not returned to the home cage, but held in a temporary cage until all testing was done for the day. For the Novel Object training day and test day, the position of each object was alternated between mice to eliminate any location bias. All movement was recorded via video camera and analyzed using Anymaze Software. All Anymaze analyses were performed using identical parameters and in a blinded manner.

### Immunofluorescent Microscopy

At the indicated day post infection, mice were anesthetized with ketamine and perfused with ice-cold PBS, followed by 4% PFA-PBS. The indicated tissues were removed and placed in 4% PFA-PBS for 24 hours. Tissues were washed 3X with PBS, then placed in 30% Sucrose-PBS for a minimum of 72 hours. Tissue was flash-frozen in OCT compound (Fisher Scientific, #23–730-571) and sliced into 10 uM thick sections using a cryostat and mounted on SuperFrost Plus Slides (Fisher Scientific, # 12–550-15). Unless otherwise indicated below, staining was performed by first blocking tissues with 5% Goat or Donkey serum and 0.1% Triton-X-100 in PBS for 1 hour at room temperature (RT). Slides were incubated with the indicated primary antibody at 4°C, overnight. After washing 3X with PBS, slides were incubated with the appropriate secondary antibody for 1 hour at RT. Slides were washed 3X with PBS, then counterstained with DAPI for 5 min at RT. Slides were coverslipped using ProLong Gold Antifade Mountant (ThermoFisher, #P36930). For IL-1β staining, antigen retrieval was performed using sodium citrate buffer (10 mM sodium citrate, 0.05% Tween 20, pH=6.0) for 10 minutes at 90°C. Slides were washed 3X with PBS then blocking was performed with a solution of 10% Donkey serum, 0.5% Bovine Serum Albumin (BSA), and 0.3% Triton-X-100. Antibody staining was performed as described above. TUNEL staining was performed using the In Situ Cell Death Detection, TMR Red kit as per the manufacturer’s instructions (Roche). Images were acquired on a AxioScanner 7 slide scanner, AxioImager epifluorescent microscope, or a Zeiss LSM 880 confocal laser scanning microscope and processed using Zeiss software. The following antibodies were used: NeuN (1:200; Cell Signaling, cat no. 12943S, clone D3S3I), BrdU (1:250; Abcam, cat. no. ab1893, polyclonal), doublecortin (1:200; Cell Signaling, cat. no. 4604S, polyclonal), GFAP (1:200; Thermo, cat. no. 13–0300, clone 2.2B10), IL-1β (1:100; R&D, cat. no. AF-401, polyclonal), IBA1 (1:400; Synaptic Systems, cat. no. 234006, polyclonal), Homer (1:200; Synaptic Systems, cat. no. 160002, polycloncal), synaptophysin (1:250; Synaptic Systems, cat. no. 101004, polyclonal), Ki67 (1:400, ThermoFisher, #14–5698-82, SolA15), Tmem119 (1:200, #PA5–119902, polyclonal), TBR2/Eomes (Abcam, #ab23345, polycolonal). Secondary antibodies to Alexa-488, Alexa-555, or Alexa-647 (Invitrogen, polyclonal) were used at a 1:500 dilution.

### BrdU Labelling

To perform *in vivo* BrdU labelling, mice were injected intraperitoneally (I.P.) every 12 hours for 2.5 days with 50 mg/kg of BrdU (Sigma Aldrich, #B5002). To visualize BrdU accumulation in tissue slices, tissue was prepared as described above in the immunofluorescent microscopy methods. Tissue sections were incubated for 5 minutes in distilled water. DNA was denatured by incubating sections in ice-cold 1N HCL for 10 min at 4°C, followed by incubation in 2N HCL at 37°C for 30 min. Acid was neutralized by washing sections in 0.1M borate buffer twice, followed by 3X washes in PBS. Slides were blocked with 5% Donkey serum, 1% BSA, and 0.1% Triton X 100 in PBS for 1 hour at RT. After blocking, slides were washed 1X with PBS then incubated with Sheep anti-BrdU (1:250) overnight at 4°C. Staining was continued as described above.

### Image Analysis

All image acquisition and analysis were performed blinded. For image quantification, a minimum of 2–3 sections per animal were averaged to obtain one biological replicate. If images were taken at 40X or above a minimum of two fields of view/ section was taken. For percent area measurements, blinded images were thresholded to the same value for each channel in Image J and measured. For cell number quantification (Ki67, DCX, TBR2, BrdU, GFAP, NeuN); In Image J, images were set to the same contrast settings and the number of positive cells was manually counted by a blinded individual. Cell number was divided by the length of the SGZ of the DG as measured via arbitrary units (pixels × 100) for each image. 3–4 sections were quantified per animal. For synapse quantification: All images were thresholded to the same value for each channel and the Spots function in Imaris was used to count the number of 3D puncta for each 5-plane z-stack. Overlapping puncta were defined as spots that were <0.1 uM apart. A minimum of 2 images/ section and 3 sections/ animal were counted.

### Flow Cytometry

Mice were euthanized via ketamine at the indicated day post infection and perfused with ice-cold PBS. Blood was collected via cardiac-puncture prior to perfusion into a tube pre-filled with 0.1mM EDTA-PBS (ThermoFisher). The indicated tissues were collected into 2 mL tubes containing 1% FBS-PBS, weighed, and stored on ice until ready for downstream processing. Lungs were processed to a single cell suspension as previously described^37^. Blood was lysed in a 5X volume of ACK lysis buffer (ThermoFisher, #A1049201) for 10 minutes on ice. Lysis reaction was stopped with 1% FBS-PBS and samples were spun down (5 minutes, 1250 rpm), passed through a 70 μM filter, and resuspended in FACS buffer (1% FBS-PBS). Brain tissue (cortex, hippocampus, or forebrain) were minced with scissors, then gently triturated using a 5 mL followed by a 1 mL pipette. The back of a 3 mL plunger was used to push tissue through a 70 uM filter. Brain single cell suspensions were spun through a 30% Percoll-PBS gradient (2000 rpm, 20 min, 4C) (Sigma-Aldrich) and the myelin debris layer was removed. Cell suspensions were resuspended in FACS buffer. All cells were incubated in TruStainFcX (Biolegend, #101319) and Live-Dead Ghost dye Violet 510 (CST, #59863S) for 30 minutes at 4°C, followed by incubation with the indicated antibodies for another 30 minutes at 4°C. Cells were washed 2X in FACS buffer, then fixed in 2% PFA-PBS for 30 minutes at RT, and removed from the BSL-3. Cells were washed 1X with FACS buffer and resuspended in FACS buffer supplemented with Precision Count Beads (Biolegend, #424902). Cells were run on a BD Fortessa at the WUSTL ChiiPs core within 48 hours of fixation. Cell counts per sample were obtained according to the Precision count beads Manufacturer’s instruction, then divided by the tissue weight to obtain the count/ gram of tissue. All data was analyzed using FlowJo version 10.8. The following antibodies were used in this study: AF700-Ly6C (Biolegend, #128023), PE-Cy7: CD3 (Biolegend, #100319), BV605: CD19 (Biolegend, #115539), BUV737:Ly6G (Thermo Fisher, #367–9668-80), APC/Cy7:I-A/I-E (Biolegend, 107627), PE:P2RY12 (Biolegend, #848004), BV421:CD11c (Biolegend, #117329), PE/Dazzle594:CD11b (Biolegend, #101255), APC:CD45

### Vaccination

The chimpanzee adenovirus-vectored vaccine encoding a prefusion stabilized spike protein (ChAd-SARS-CoV-2-S) from the original Wuhan variant and the empty adenovirus vector control (ChAd-CTL) was originally described in Hassan et al., 2020^45^. Stock vaccine and control was diluted in PBS to a working concentration of 2.5 × 10^9^ viral particles/mL. Mice were anesthetized with ketamine and intranasally inoculated with 1 × 10^8^ viral particles of ChAd-S or ChAd-CTL. At 21 days post vaccination, cheek bleeds were performed to collect sera and FRNTs were performed on sera from control and vaccinated mice to measure neutralizing antibody titers as previously described^63^. At day 30 post vaccination, mice were infected with B.1.351 or mock infected as described above in the Infections methods.

### Statistical Analysis

All experiments were repeated a minimum of twice. For flow cytometry, viral titer, immunofluorescent microscopy, qRT-PCR experiments a minimum of 4–6 animals per group were used. For behavior experiments, weight loss analysis, and survival curves a minimum of 8 animals per group was used. All statistical analysis was performed in GraphPad Prism v9 with the appropriate test for the indicated analysis. The following statistical tests were used in this study: Student’s t test, unpaired one- or unpaired or paired two-way analysis of variance (ANOVA), and simple linear regressions. Throughout a manuscript, a result was not considered significant unless a p-value less than 0.05 was achieved.
